# Improvement of acetate tolerance of *Escherichia coli* by introducing the PHB mobilization pathway

**DOI:** 10.1128/aem.02454-24

**Published:** 2025-04-04

**Authors:** Dong Meng, Shuai Wang, Ke Zhao, Yan Luo, Xu Li, Ying Wang

**Affiliations:** 1 Key Laboratory of Medical Molecule Science and Pharmaceutical Engineering, Ministry of Industry and Information Technology, Institute of Biochemical Engineering, Department of Chemical Engineering, School of Chemistry and Chemical Engineering, Beijing Institute of Technology 504823 , Beijing, China; 2 MOE Key Laboratory of Cluster Science, Beijing Key Laboratory of Photoelectronic/Electrophotonic Conversion Materials, School of Chemistry and Chemical Engineering, Beijing Institute of Technology 624358 , Beijing, China; Shanghai Jiao Tong University, Shanghai, China

**Keywords:** PHB mobilization, acetate stress, membrane, *Escherichia coli*, transcriptomics

## Abstract

Poly-β-hydroxybutyrate (PHB) mobilization has been recognized as an effective measure in bacteria for host survival under stressful conditions. Here, we report that PHB mobilization is also involved in improving *Escherichia coli* resistance to acetic acid stress. Under 0.06% (vol/vol) acetic acid stress, the cell viability of strain M5 with PHB mobilization reached 52.8%, significantly higher than the 25.2% observed for strain M1 without PHB mobilization. Comparison of transcriptome data between M1 and M5 strains identified genes associated with membrane formation that participate in acetic acid tolerance. As a result, the membrane integrity of the M5 strain was significantly increased by 31.5% compared to strain M1. According to physiological membrane analysis, strain M5 showed a 35.3% increase in the ratio of cyclic to unsaturated fatty acids compared to strain M1 under 0.06% (vol/vol) acetic acid. These results indicate that PHB mobilization can regulate membrane components, consequently enhancing cell tolerance to acetic acid. Furthermore, acetate serves as a cost-effective alternative carbon source for microbial cultivation. Then, the succinate-producing strain M8 containing PHB mobilization was used to produce succinate and PHB from sodium acetate. Interestingly, PHB mobilization improved sodium acetate tolerance and utilization in *E. coli*, while enhancing succinate and PHB production. Finally, strain M8 can accumulate 23.93 g/L succinate and 7.21 g/L PHB using sodium acetate under fed-batch fermentation. In conclusion, this work reveals the role of PHB mobilization under acetate stress and provides a basis for acetate utilization.

**IMPORTANCE:**

This study investigated the underlying mechanism through which PHB mobilization enhances *Escherichia coli* tolerance to acetic acid stress. PHB mobilization improved *E. coli* tolerance to acetic acid, leading to enhanced cell viability. The transcriptome results indicated that PHB mobilization mainly alters the expression of membrane-associated genes, such as gene *Bhsa* (encoding outer membrane protein), leading to increased resistance to acetic acid. The membrane physiological analysis indicated that PHB mobilization plays a critical role in membrane integrity, fluidity, and lipid components under acetic acid stress. Moreover, we proposed a novel approach for the co-synthesis of succinate and PHB in recombinant *E. coli* from sodium acetate. The succinate-producing strain M8 harboring PHB mobilization can efficiently co-produce succinate and PHB, exhibiting better cell growth and sodium acetate utilization compared to the control strain without PHB mobilization. These findings indicate that PHB mobilization has implications for developing robust *E. coli* and their biosynthesis applications.

## INTRODUCTION

Bacteria are widely distributed in nature and actively participate in the process of recycling materials. Bacterial cells exist in volatile environmental conditions, such as changes in temperature, pH, nutrient availability, and osmotic pressure (1). To ensure cell survival, bacteria have evolved regulatory mechanisms to respond to environmental changes, and this process involves complex gene regulation (2). Among them, protective molecule synthesis metabolism is a common mechanism that ensures cell viability. Under nutritious conditions, bacteria can produce intracellular polymers (such as glycogen, polyphosphate, and polyhydroxyalkanoate [PHA]) as a reserve. These polymers are usually further utilized by the bacteria themselves, enabling cells to cope with extreme conditions (3).

Poly(3-hydroxybutyrate) (PHB), the most frequent type in the polyhydroxyalkanoate family, is an energy storage polymer produced by numerous prokaryotes in the form of intracellular granules (4). The PHB mobilization, consisting of biosynthesis and degradation, is a cyclic mechanism that has been found in many bacteria, and the metabolic pathway involves four genes, including beta-ketothiolase (*phaA*), acetoacetyl-CoA reductase (*phaB*), PHA synthase (*phaC*), and PHA depolymerase (*phaZ*) (5). In recent years, the biosynthesis of PHB has received extensive attention. The polymers have excellent biocompatibility and biodegradability, and they are recognized as a green, environmentally friendly material (6). In addition to applied value, their biological function is far more comprehensive. The presence of PHB mobilization in bacterial cells has been shown to improve the resistance of cells against various stress factors (7). In all kinds of stress, acid stress is widely encountered in microbial growth, stemming from natural environments or production activities (e.g., utilization of cheap acetate substrates). Consequently, organisms must adapt to stress environments to support their essential biological functions (8). Besides, excellent acetate tolerance presents a highly advantageous phenotypic attribute for microorganisms employed in the domain of industrial biotechnology, notably in the context of the utilization of sustainable feedstocks, such as excess sludge of wastewater and lignocellulosic biomass (9).

In recent years, engineered *Escherichia coli* already holds significant potential for the synthesis of proteins, organic acids, polysaccharides, and valuable bioproducts (10). As energy and environmental challenges persistently grow, the utilization of cost-effective acetate by *E. coli* to produce high-value products has become an area of significant interest (11). Acetate, including sodium acetate and acetic acid, can be produced by adjusting the pH and utilized by *E. coli* (12). Numerous studies have reported the use of sodium acetate by engineered *E. coli* for the synthesis of various chemicals. However, the toxicity of acetate reduces the biosynthetic efficiency of *E. coli*, and research aimed at enhancing the acetate resistance of *E. coli* and elucidating its underlying mechanisms remains relatively constrained. Therefore, given the physiological role of PHB mobilization in bacteria, we wondered whether the introduction of PHB mobilization would have unexpected effects on acetate tolerance and utilization.

In this study, we explored the impact of introducing the PHB mobilization pathway on enhancing cell survival in the presence of acetate stress. The transcriptome sequencing was performed to elucidate the role of PHB mobilization in the response to acetic acid stress perturbation. This work highlights the significant role of PHB mobilization in enhancing resistance to acetic acid stress. The introduction of PHB mobilization changes membrane lipid composition, improving cell membrane integrity and increasing acid tolerance. Interestingly, PHB mobilization in *E. coli* can also enhance resistance and utilization of sodium acetate, promoting succinate and PHB co-production from sodium acetate. This study provides a novel insight into the physiological function and application of PHB mobilization in bacteria.

## RESULTS

### Protective effect of PHB mobilization in *E. coli* against acetic acid stress

**Fig 1:**
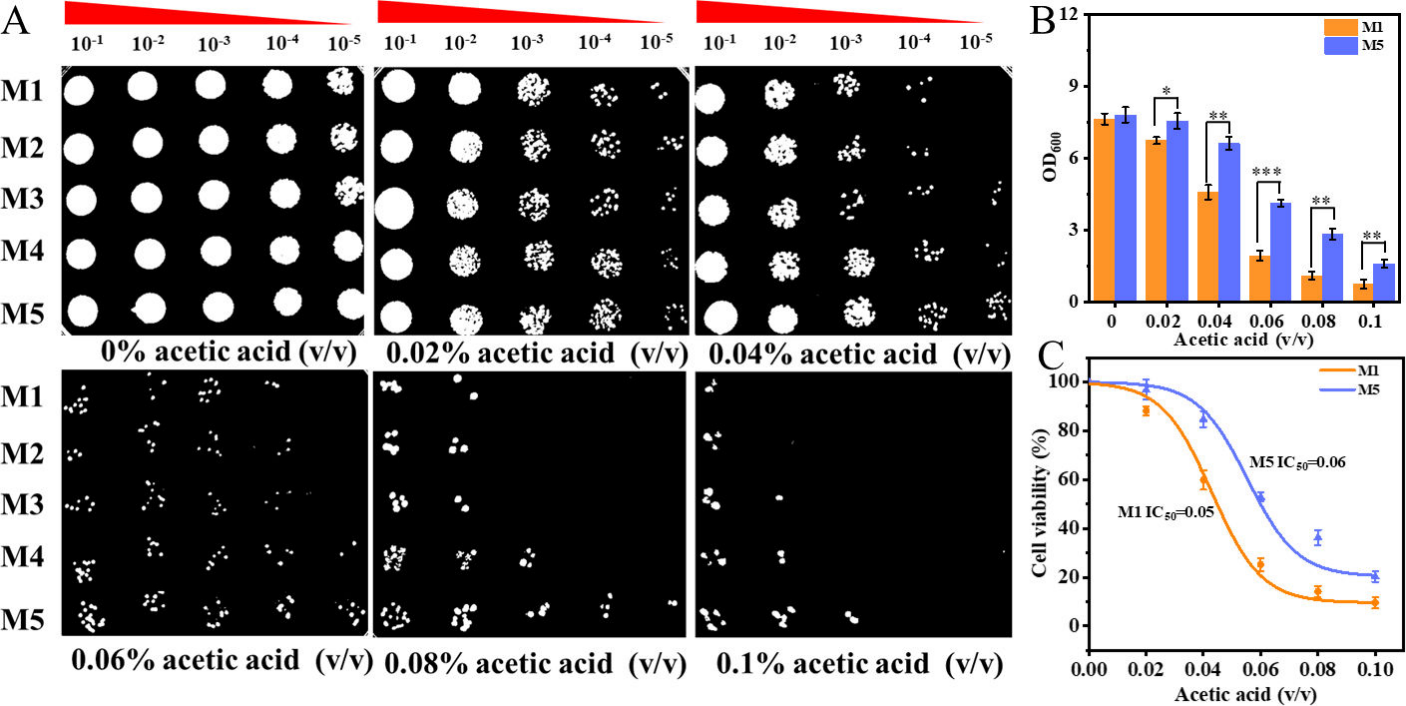
PHB mobilization is essential for cell growth under acetic acid stress. (**A**) Growth status of the parent strains (M1), M2, M3, M4, and M5 on LB plates at different concentrations of acetic acid. (**B**) The final optical density (OD)_600_ of strains M1 and M5 after 48 hours at 0% and 0.06% (vol/vol) acetic acid. (**C**) IC50 of strains M1 and M5 at different concentrations of acetic acid. The data refer to biological repeats; the error bars represent standard deviations (SD). *P ≤ 0.05, **P ≤ 0.01, and ***P ≤ 0.001.

To achieve the metabolic cycle of PHB in *E. coli*, we constructed an engineered strain by introducing a complete PHB mobilization pathway induced by stress promoter rpoS. To determine the critical role of PHB mobilization in *E. coli* cell growth under acetic acid stress, we first carried out spot assays of parental strains M1 (puc19), M2 (puc19-*phaA*), M3 (puc19-*phaAB*), M4 (puc19-*phaCAB*), and M5 (puc19-*phaCABZ*) grown on LB medium at 0%–0.1% (vol/vol) acetic acid. As shown in Fig. 1A, the M5 strain containing PHB mobilization genes showed the most significant growth improvement at 0.06% (vol/vol) acetic acid, whereas no significant difference in cell growth was observed in strains without PHB mobilization.

According to optical density (OD_600_) measurement analysis, the final OD_600_ value of strains M1 and M5 gradually decreased with the addition of acetic acid (Fig. 1B). At 0% (vol/vol) acetic acid, both strains showed equivalent growth performance. At 0.06% (vol/vol) acetic acid, strain M1 exhibited a 74.8% reduction in final OD_600_, whereas the final OD_600_ of strain M5 was 2.1 times greater than that of M1. Furthermore, at 0.06% (vol/vol) acetic acid, the cell viability of strains M1 and M5 was 25.2% and 52.8%, respectively, and half-maximal inhibitory concentration (IC_50_) values were 0.05 and 0.06 (vol/vol) acetic acid, respectively (Fig. 1C). Thus, these results demonstrated that the introduction of PHB mobilization can significantly improve the growth of the cells under high acetic acid concentration.

In order to track the mobilization of PHB under acetic acid stress, the Nile red dyeing method was used to characterize PHB based on a previous study (13). Fluorescent staining results of strain M5 showed that the intensity of red fluorescence decreased after adding acetic acid for 2 hours (Fig. S1A and B). Moreover, we also detected the presence of (R)-3-hydroxybutyric acid (3HB) in the culture supernatant (Fig. S1C). Therefore, the above observations suggested that the PHB has undergone degradation. In addition, to determine whether 3HB contributes to resistance against acetic acid stress, the investigation was tested by adding increasing quantities of 3HB to strain M1. As shown in Fig. S1D, the final biomass values of M1 gradually increase with the rising 3HB concentration. However, continual supplementation with 3HB does not invariably result in sustained increases in biomass, and the maximum final biomass value increased by 74.3% at 20 mM compared to 0 mM 3HB. Obviously, this value remains 13.9% lower than that of the M5 strain, indicating that the sole addition of 3HB cannot fully substitute for PHB mobilization. Additionally, to determine whether the expression of the enzymes corresponding to genes *phaCABZ* affects acetic acid tolerance in *E. coli*, we constructed four enzyme-inactive (PhaA_C88S_, PhaB_D94A_, PhaC_T323I_, and PhaZ_C183A_) mutants (strain M5-2) based on previous reports (14–17). Strain M5-2 was compared with strain M5, which expresses the active enzymes, to assess the specific contribution of PHB mobilization to acetic acid tolerance. First, the synthesis of PHB and 3HB under acetic acid stress was assessed in strain M5-2. As shown in Fig. S1E, no synthesis of PHB or 3HB was detected in strain M5-2, whereas strain M5 produced PHB and 3HB at concentrations of 0.84 and 0.27 g/L, respectively. Moreover, the growth of the three strains did not differ significantly under conditions without acetic acid stress. However, under acetic acid stress, the growth of strains M1 and M5-2 was significantly reduced, decreasing by 53.3% and 52.7% compared with strain M5, respectively (Fig. S1F). This suggested that the enzymes encoded by genes *phaCABZ* have no impact on *E. coli*’s resistance to acetic acid stress. In conclusion, these results demonstrated that PHB mobilization appears to play a crucial role in the growth of *E. coli* under acetic acid stress.

### Global transcriptome analysis of strains M1 and M5 under acetic acid stress

**Fig 2:**
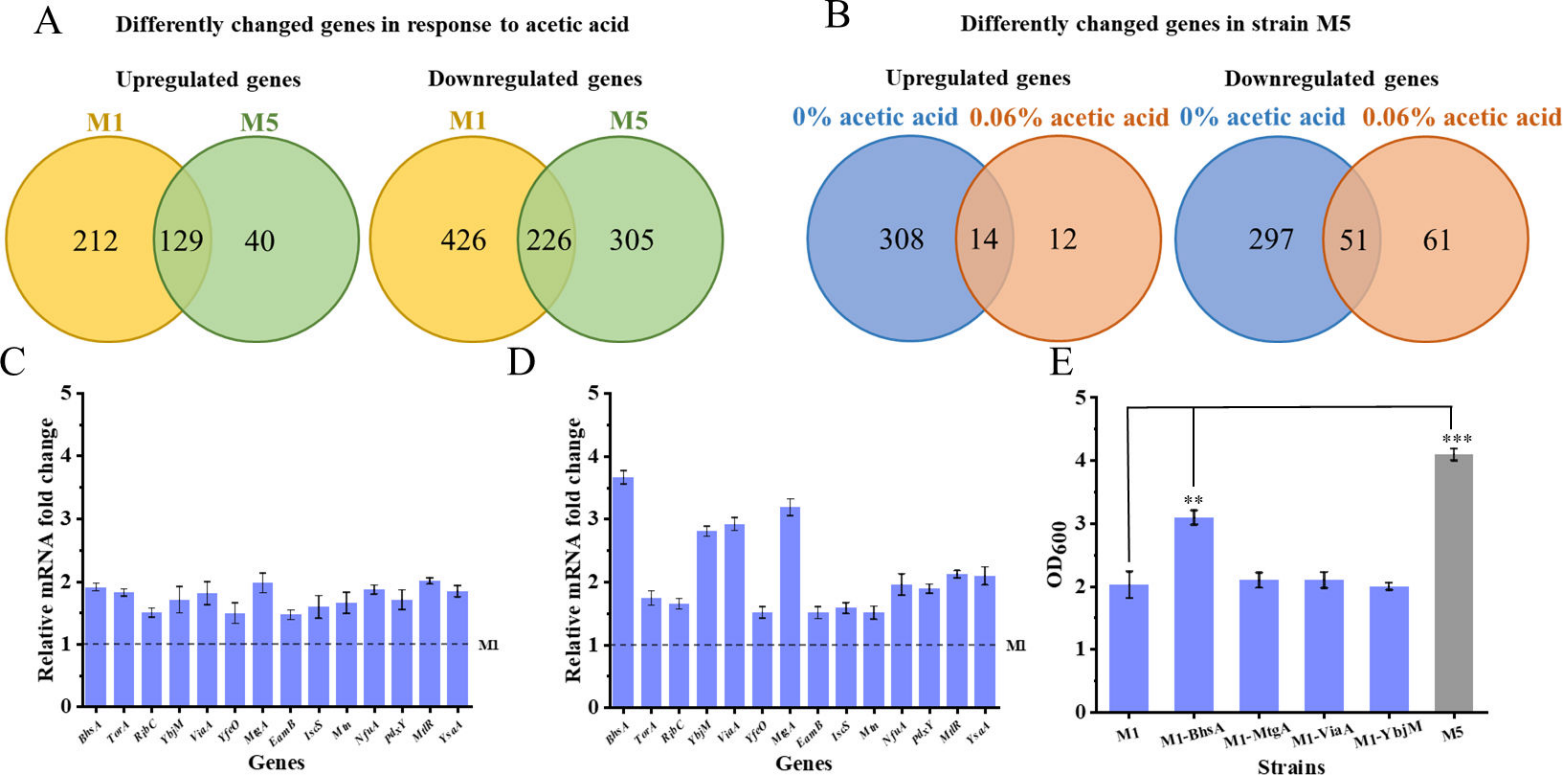
Global transcriptome analysis of strains M1 and M5. (**A**) Venn diagram depicts the overlap of upregulated and downregulated genes between strains M1 and M5 at 0% compared with 0.06% acetic acid. (**B**) Venn diagram depicts the overlap of upregulated and downregulated genes between 0% and 0.06% (vol/vol) acetic acid in strain M5 compared with strain M1. (**C and D**) mRNA levels of commonly upregulated 14 genes in strain M5 relative to those of strain M1 at 0% and 0.06% (vol/vol) acetic acid. (**E**) The most upregulated genes were overexpressed, and the mutant strains were cultured in LB medium at 0.06% (vol/vol) acetic acid. *P ≤ 0.05, **P ≤ 0.01, and ***P ≤ 0.001.

To gain further insight into the physiological role of PHB mobilization in *E. coli* resisting acetic acid stress, transcriptome sequencing (RNA-seq) analysis was performed to determine gene expression levels in strains M1 and M5 at 0% and 0.06% (vol/vol) acetic acid. Then, significantly expressed genes were screened using restrictive thresholds. We first compared the gene expression of strain M5 with or without 0.06% (vol/vol) acetic acid addition. Transcriptome analysis indicated that the expression of 700 genes was significantly altered in strain M5, including 169 upregulated genes and 531 downregulated genes. As for strain M1, 993 genes showed significant changes, of which 341 were upregulated and 652 were downregulated (Fig. 2A). Moreover, strains M1 and M5 share 129 upregulated genes and 226 downregulated genes. Gene Ontology (GO) analysis revealed that the commonly upregulated genes are mainly related to lipid modification (GO:0030258), peptidoglycan metabolism (GO:0000270), response to pH (GO:0009268), and carbohydrate metabolism (GO:0005975), Besides, the commonly downregulated genes are primarily involved in transmembrane transport (GO:0055085), fatty acid metabolism (GO:0046459), biofilm formation (GO:0044010), and cellular response to DNA damage stimulus (GO:0006974).

Transcriptome results also showed that the expression of 322 genes was upregulated and the expression of 348 genes was downregulated in strain M5 compared to the same genes’ expression in strain M1 at 0% acetic acid. However, when the cells were treated with 0.06% (vol/vol) acetic acid, strain M5 contained 26 upregulated genes and 112 downregulated genes compared to M1. Moreover, it also revealed that the 14 upregulated genes and 51 downregulated genes were common to both the normal and acetic acid stress conditions (Fig. 2B). According to GO analysis, the results indicated that the upregulated genes shared between both conditions were implicated in biofilm formation (GO: 0044010), transmembrane transport (GO:1903712), protein folding (GO:0006457), cell wall biogenesis (GO:0043164), negative regulation of transcription (GO:0045892), and riboflavin biosynthesis (GO:0009231). The commonly downregulated genes were predominantly associated with various processes, such as DNA replication (GO:0006260), transmembrane transport (GO:0055085), and signal transduction (GO:0007165). Moreover, the analysis also indicated that membrane transport was the most significantly affected pathway (as mapped in the KEGG database), accounting for 17.5% of all affected genes in strain M5 compared with levels in strain M1 at 0.06% (vol/vol) acetic acid (Fig. S2A). Figure S2B also shows significant enrichment among genes involved in membrane transport. Remarkably, the membrane transport pathway includes many membrane proteins, such as the significantly increased expression of the *Bhsa* gene, which is mainly involved in membrane formation and copper ion transport (18). The above results suggest that the introduction of PHB mobilization may affect membrane biosynthesis in response to acetic acid stress.

In addition, the mRNA levels of the 14 commonly upregulated genes were also tested at 0% and 0.06% (vol/vol) acetic acid using quantitative reverse transcription-PCR (qRT-PCR) analysis (Fig. 2C and D). At 0% (vol/vol) acetic acid, mRNA levels of *BhsA*, *TorA*, *RibC*, *YbjM*, *ViaA*, *YfeO*, *MtgA*, *EamB*, *IscS*, *Mtn*, *NfuA*, *PdxY*, *MtlR*, and *YsaA* in strain M5 increased by 1.9-, 1.8-, 1.5-, 1.7-, 1.8-, 1.5-, 2.0-, 1.5-, 1.6-,1.7-, 1.9-, 1.7-, 2.0-, and 1.8-fold, respectively, compared with the corresponding values of strain M1. At 0.06% (vol/vol) acetic acid, mRNA levels of *BhsA*, *TorA*, *RibC*, *YbjM*, *ViaA*, *YfeO*, *MtgA*, *EamB*, *IscS*, *Mtn*, *NfuA*, *PdxY*, *MtlR*, and *YsaA* increased by 3.7-, 1.7-, 1.7-, 2.8-, 2.9-, 1.5-, 3.2-, 1.5-, 1.6-, 1.5-, 2.0-, 1.9-, 2.1-, and 2.0-fold, respectively. It can be found that the four genes *Bhsa*, *YbjM*, *ViaA*, and *MtgA* are most significantly upregulated by comparing the above results. Furthermore, the four genes mentioned above were overexpressed in strain M1 separately, and the resulting effect on resistance to acetic acid was assessed. Interestingly, only the overexpression of *Bhsa* can improve the resistance to acetic acid stress. At 0.06% (vol/vol) acetic acid, the final biomass of M1-*Bhsa* increased by 52.9% compared with the corresponding value of the control strain M1, but it was still lower than that of strain M5 (Fig. 2E). Notably, the gene *Bhsa* is involved in regulating cell membrane formation of *E. coli* as mentioned in the above paragraph. In short, PHB mobilization might regulate cell membrane physiology in response to acetic acid stress conditions.

### PHB mobilization affects membrane functions in *E. coli* under acetic acid stress

**Fig 3:**
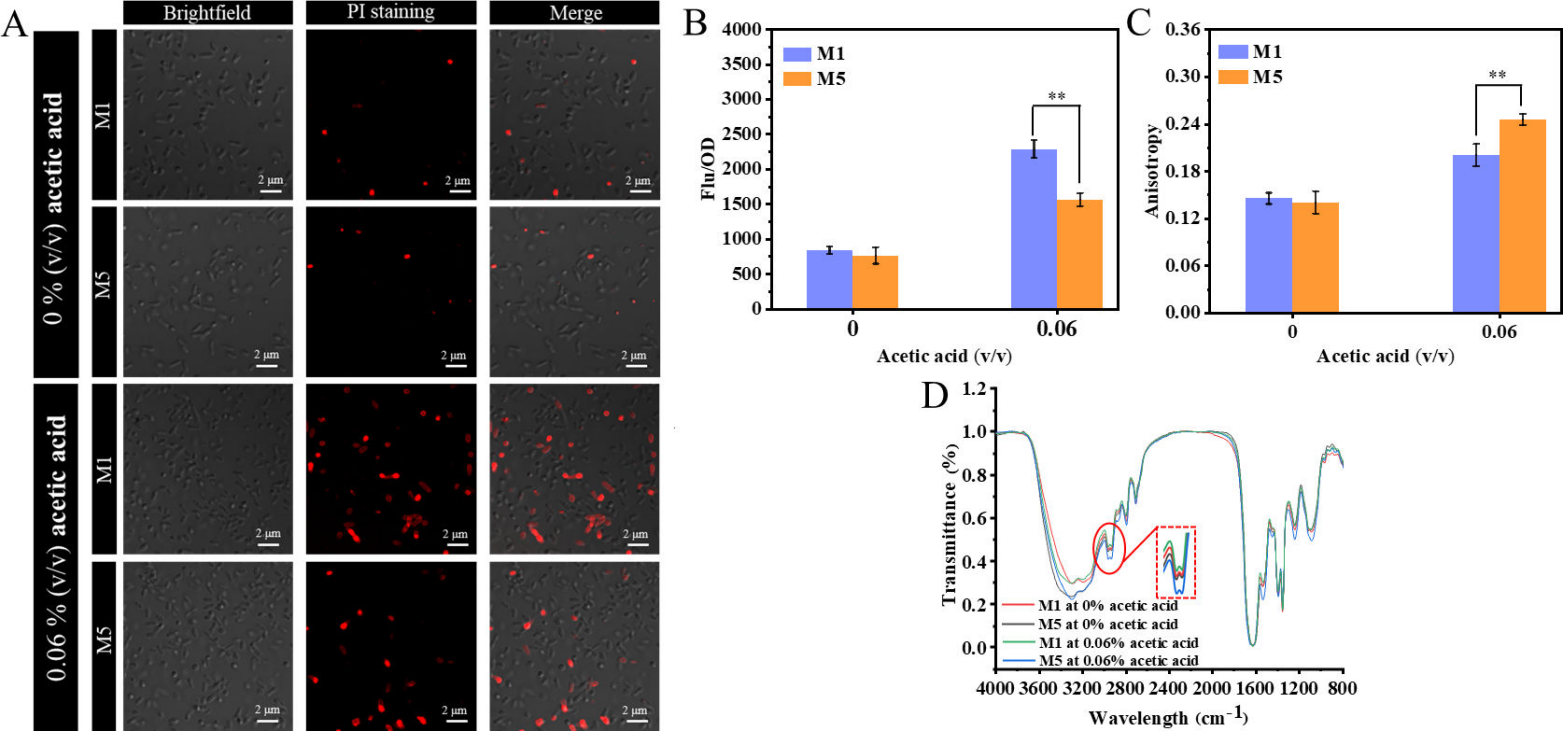
Analysis of membrane integrity, fluidity, and composition change. (**A**) Fluorescent microscopy analysis of membrane integrity in strains M1 and M5 at 0% and 0.06% (vol/vol) acetic acid. (**B**) PI uptake of strains M1 and M5 at 0% and 0.06% (vol/vol) acetic acid. (**C**) Anisotropy values of strains M1 and M5 at 0% and 0.06% (vol/vol) acetic acid. (**D**) FTIR spectra of strains M1 and M5 at 0% and 0.06% (vol/vol) acetic acid. *P ≤ 0.05, **P ≤ 0.01, and ***P ≤ 0.001.

To investigate the effect of PHB mobilization on cell membrane, cells of strains M1 and M5 were subjected to 0% and 0.06% (vol/vol) acetic acid conditions. First, the membrane integrity was evaluated based on propidium iodide (PI) staining. At 0% (vol/vol) acetic acid, the two strains exhibited similar PI uptake. However, when the cells were exposed to acetic acid, the PI uptake of both strains was significantly improved. Notably, the PI uptake of strain M5 cells was significantly reduced at 0.06% (vol/vol) acetic acid, with a 31.5% drop compared to strain M1 (Fig. 3B). Similarly, fluorescence microscope analysis showed agreement with the above results. At 0% (vol/vol) acetic acid, the fluorescence microscope analysis indicated that the majority of cells from both strains M1 and M5 displayed intact membranes, whereas at 0.06% (vol/vol) acetic acid, strain M5 had more intact cells than strain M1 (Fig. 3A).

Furthermore, we also evaluated membrane fluidity, which is an important parameter reflecting cell membrane physiological function (19). As illustrated in Fig. 3C, the membrane fluidity of strains M1 and M5 decreased significantly with acetic acid treatment. However, in response to acetic acid stress, strain M5 showed a 22.5% reduction in membrane fluidity compared to strain M1. The previous study indicated that the adjustment of membrane integrity and fluidity usually results in changes in membrane composition (20). Thus, the Fourier transform infrared (FTIR) spectroscopy analysis was used to detect the structural changes of bacterial cell membranes under acetic acid stress. As shown in Fig. 3D, the intense absorption peak at approximately 2,930 and 2,850 cm^−1^ corresponds to the CH_2_ antisymmetric stretching and CH_2_ symmetric stretching of fatty acids, which is consistent with a previous study (21). Notably, the obvious increases occurred in a region of fatty acids in strain M5 with 0.06% (vol/vol) acetic acid treatment, and these increases suggested a rise in membrane fatty acid content. The result was also confirmed by the increase in the intensity of another fatty acid-related band (CH_2_ bending) at around 1,450 cm^−1^. In a word, strain M5 with PHB mobilization may resist acetic acid stress mainly by altering fatty acid composition.

According to the above results, we further determined fatty acid compositions of the M5 and M1 strains at 0% and 0.06% (vol/vol) acetic acid. The analysis results showed that all recombinant *E. coli* had eight types of fatty acid, which can be divided into saturated fatty acids (SFAs), unsaturated fatty acids (UFAs), and cyclic fatty acids (CFAs), and the results were consistent with those previously reported (Table 1) (22). At 0% (vol/vol) acetic acid, the proportions of the above fatty acids in strains M1 and M5 were basically similar. However, at 0.06% (vol/vol) acetic acid, the UFA proportion of the two strains exhibited an obvious reduction, while the 42.2% reduction of strain M5 was lower than strain M1 (25.0%). Besides, the CFA proportion showed obvious improvement, the two strains increased by 15.4% (M1) and 23.9% (M5), respectively. At 0.06% (vol/vol) acetic acid, the ratio of cyclic to unsaturated fatty acids of strain M5 increased by 35.3% compared with strain M1. In conclusion, the above results suggested that the membrane function in the M5 strain is regulated to resist acetic acid stress by changing the fatty acid content.

**TABLE 1:** Variation in the percentage of fatty acids within strains M1 and M5 across different concentrations of acetic acid

Fatty acids	Total composition (%) at various concentrations of acetic acid			
	0% (M1)	0% (M5)	0.06% (M1)	0.06% (M5)
SFAs				
C12:0	3.3 ± 0.2	3.5 ± 0.1	5.3 ± 0.2	4.8 ± 0.2
C14:0	5.3 ± 0.2	5.2 ± 0.1	5.6 ± 0.2	6.6 ± 0.2
C16:0	37.2 ± 0.5	36.3 ± 0.2	37.0 ± 0.2	38.5 ± 0.1
C18:0	13.0 ± 0.3	13.1 ± 0.3	12.8 ± 0.5	12.0 ± 0.3
Total SFAs	58.8	58.1	60.7	61.9
UFAs				
C16:1 ω7	11.1 ± 0.2	11.4 ± 0.1	8.1 ± 0.2	6.6 ± 0.3
C18:1 ω7	9.3 ± 0.2	9.7 ± 0.3	7.2 ± 0.1	5.6 ± 0.4
Total UFAs	20.4	21.1	15.3	12.2
CFAs				
C17:0 cyclo	11.2 ± 0.3	11.1 ± 0.1	13.1 ± 0.2	14.0 ± 0.2
C19:0 cyclo	9.6 ± 0.5	9.8 ± 0.4	10.9 ± 0.1	11.9 ± 0.4
Total CFAs	20.8	20.9	24	25.9

### PHB mobilization improves co-production of succinate and PHB in recombinant *E. coli* from sodium acetate

**Fig 4:**
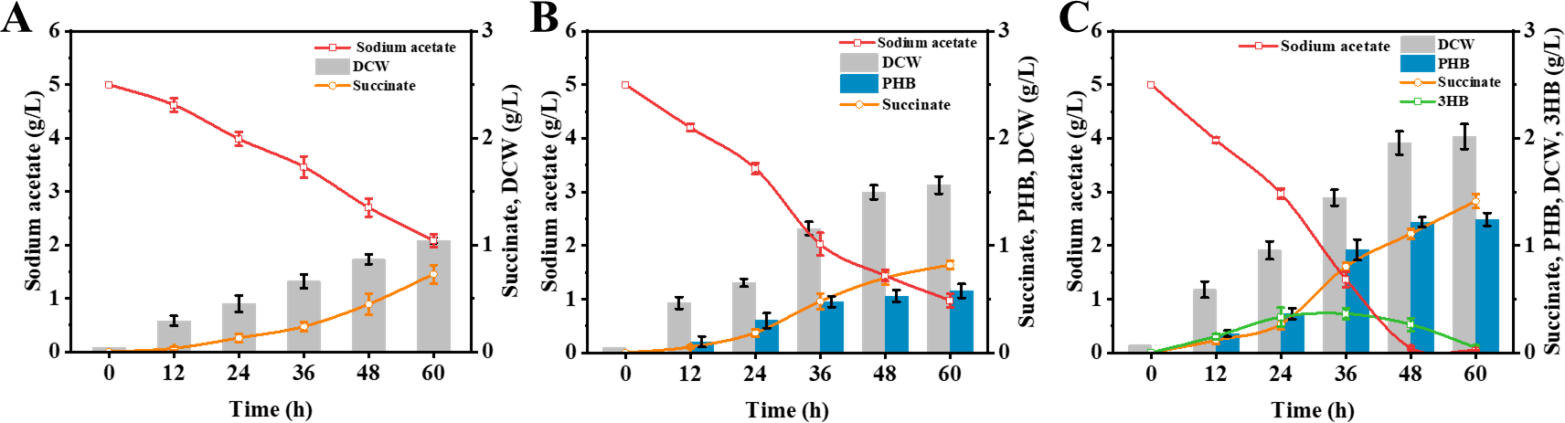
PHB mobilization promotes succinate and PHB co-production from sodium acetate. (**A**) Batch cultivation of strain M6. (**B**) Batch cultivation of strain M7. (**C**) Batch cultivation of strain M8.

**Fig 5:**
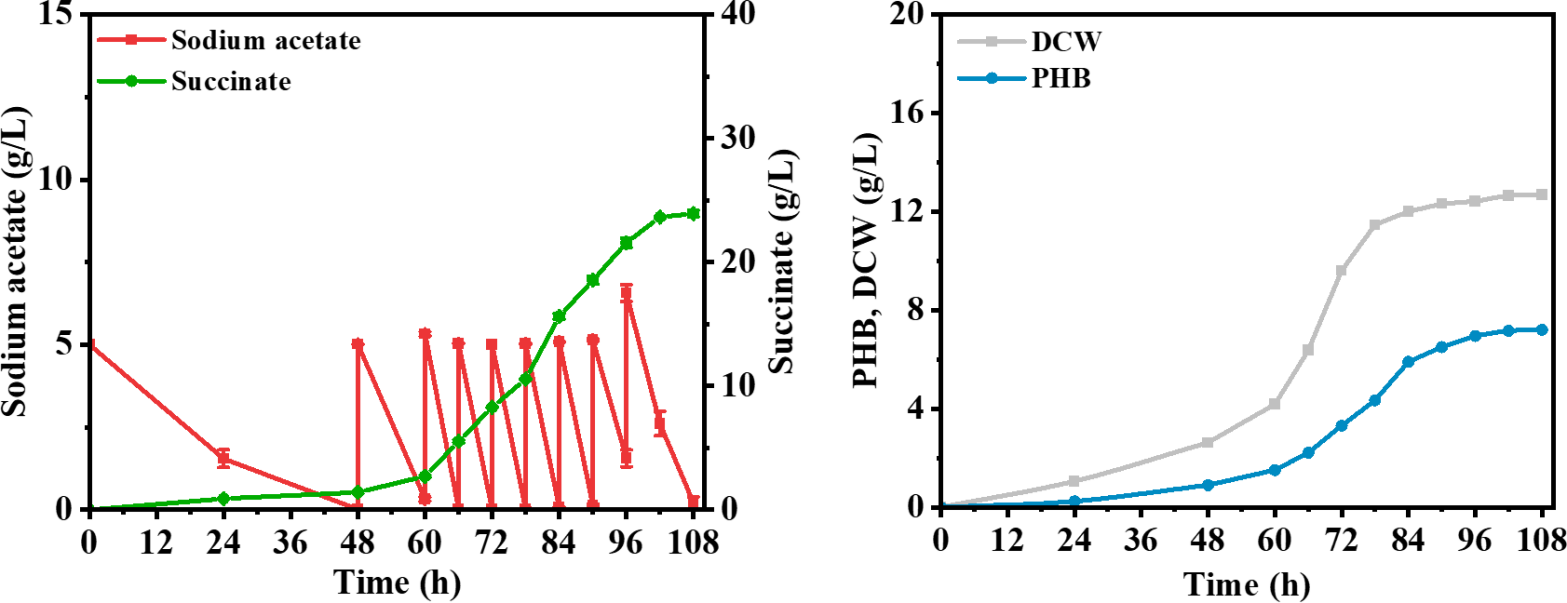
Bioreactor fed-batch fermentations for co-production of succinate and PHB in strain M8 from sodium acetate.

According to previous study, an approach for simultaneous production of succinate (extracellular) and PHB (intracellular) in *E. coli* with glucose had been developed based on the advantage of both products (23). Reducing production costs by further developing acetate as a fermentation carbon source is essential. The introduction of the PHB mobilization in *E. coli* has been determined to provide cellular resistance to acetic acid stress according to the above results, so we wondered if the introduction of PHB mobilization could enable the *E. coli* to use acetate for succinate and PHB co-production efficiently. Considering that the carbon mass from 0.06% (vol/vol) acetic acid was insufficient to support the growth and production of *E. coli*, sodium acetate was selected as the carbon source. Acetate, including sodium acetate or acetic acid, can be obtained from lignocellulose hydrolysate through simple pH adjustment, but both are toxic to *E. coli* cells (12). First, we investigated whether the introduction of the PHB mobilization pathway could enhance the resistance of *E. coli* to sodium acetate. As shown in Fig. S3, under the condition of 5 g/L sodium acetate, the final growth OD value of strain M5 was 4.56, which was 76.9% higher than that of strain M1. To assess the broader applicability of the protective effect of PHB mobilization, we tested the sensitivity of *E. coli* to various stress conditions. Interestingly, strain M5 exhibited greater resistance to HCl, lactate, and succinate compared to strain M1 (Fig. S3). To produce succinate in *E. coli*, the succinate dehydrogenase (*sdhAB*), isocitrate lyase regulator (*iclR*), and malic enzymes (*maeB*) genes were deleted in *E. coli* DH5α to generate strain M6 based on previous report (24). Then, we first made a batch cultivation in M9 medium with 5 g/L sodium acetate using strain M6. The results showed that M6 could produce 0.73 g/L succinate in batch fermentation (Fig. 4A). Then, the genes *phaCAB* and *phaCABZ* were introduced into strain M6, resulting in strains M7 and M8, respectively. As shown in Fig. 4, the final DCW value of strain M8 with sodium acetate was 2.02 g/L, which was higher than that of strains M6 (1.04 g/L) and M7 (1.56 g/L). Besides, strain M8 exhibited a stronger sodium acetate utilization ability compared with strains M6 and M7, and complete depletion of the carbon source was observed at the 60-hour mark. Moreover, strain M8 accumulated 1.41 g/L succinate, which was higher than that of strains M6 (0.73 g/L) and M7 (0.82 g/L). Meanwhile, both strains M7 and M8 can produce PHB, which gradually accumulates as the substrate is consumed. However, the PHB production of strain M8 (1.24 g/L) was higher than that of strain M7 (0.58 g/L). Obviously, strain M8 was the sole producer of 3HB. The concentration of 3HB increased concomitantly with the consumption of sodium acetate, reaching a peak of 0.36 g/L at 36 hours. Subsequently, as sodium acetate was depleted, strain M8 began to utilize 3HB, leading to a reduction in 3HB concentration. Obviously, the mobilization of PHB improved the sodium acetate utilization and cell growth of strain M8, enabling the effective implementation of a strategy for succinate and PHB co-production in *E. coli* from sodium acetate. Finally, fed-batch fermentations were conducted to evaluate the potential of strain M8 as a cell factory, and the cultivation was carried out in a 5 L bioreactor with the addition of sodium acetate. As shown in Fig. 5, strain M8 experienced a 48-hour adaptation period, during which its ability to utilize acetic acid was limited, possibly due to the toxicity of sodium acetate. With the biomass of strain M8 increased, the utilization of sodium acetate progressively accelerated. By 84 hours, the culture entered a plateau phase, characterized by a reduced rate of carbon source consumption and stabilization of biomass. After 108 hours of cultivation, the production of succinate and PHB reached 23.93 and 7.21 g/L, respectively, resulting in a total target compound yield of 0.69 g/g.

## DISCUSSION

In this study, we investigated the effect of PHA mobilization on acetate stress. Moreover, our research showed for the first time that PHA mobilization plays a pivotal role in modulating the expression of genes associated with cell membrane formation. This regulatory mechanism leads to alterations in membrane lipid composition, affecting membrane integrity and fluidity. Ultimately, this process contributes to enhanced cell growth under acetate stress conditions.

**Fig 6:**
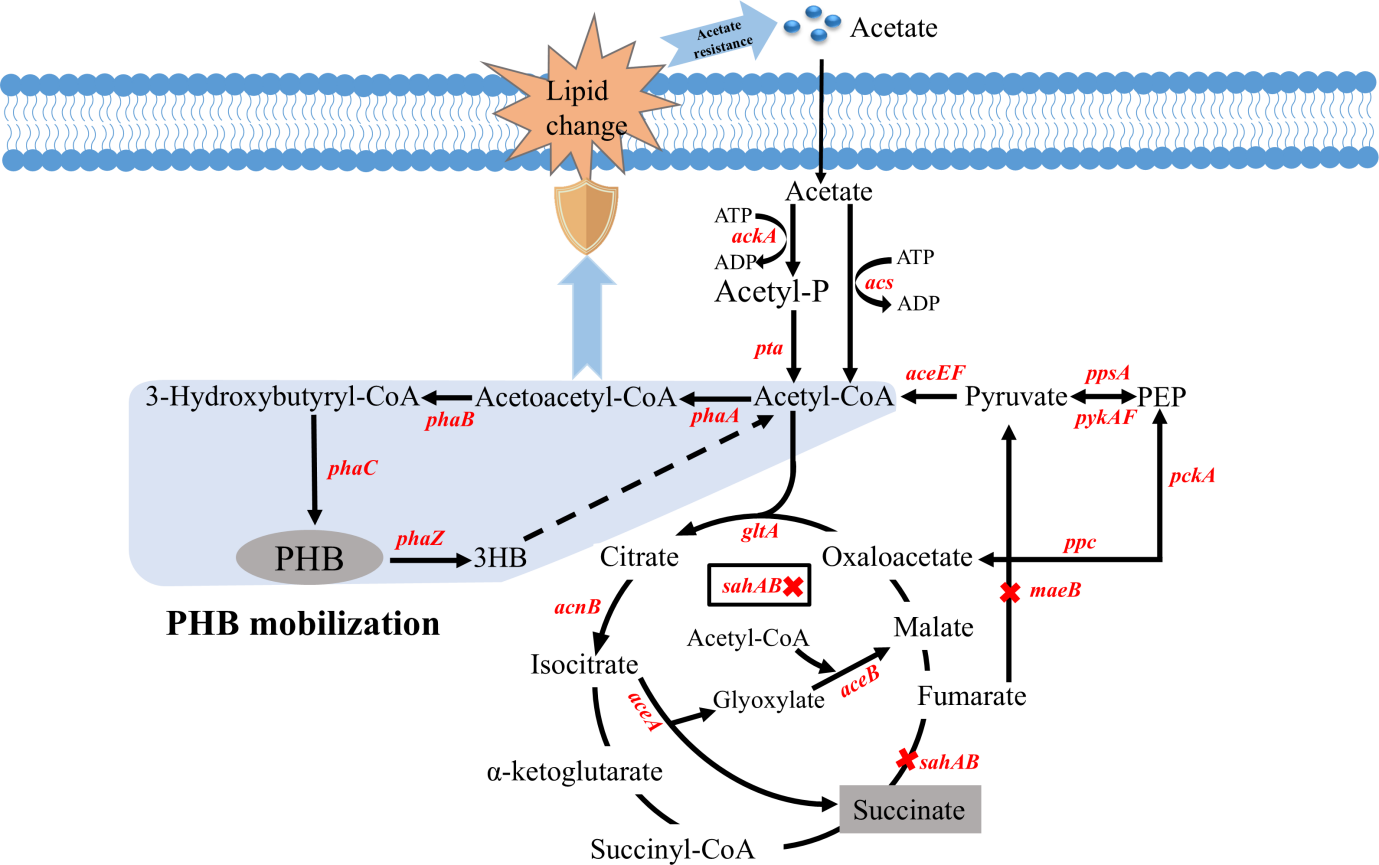
Designed metabolic pathways for the co-production of succinate and PHB in strain M8 from acetate.

In microorganisms, the process of PHA formation is crucial for survival under stress (7). PHB formation can support the survival of bacteria under various stresses, but we showed that this process is insufficient to protect bacteria from stress pressure. In this study, the pathway of PHB mobilization was introduced into *E. coli* under the promoter *rpos*, which is a pressure-responsive promoter (25). The metabolism process of PHB mobilization in *E. coli* involves four metabolites, including acetoacetyl-CoA, R-3-hydroxybutanoyl-CoA, PHB, and 3HB, which are synthesized by the *phaA*, *phaAB*, *phaCAB*, and *phaCABZ* genes, respectively (Fig. 6). Obviously, strain M5 containing the compete PHB mobilization pathway exhibited better resistance to acetic acid stress compared to engineered strains without PHB mobilization. Then, by comparing the growth of strains M5 and M5-2 under acetic acid stress, we ruled out the possibility that the proteins expressed from the *phaCABZ* genes affect acetate tolerance. Thus, we initially attributed the characteristics of the M5 strain to the release of 3HB by the mobilization of PHB, and the 3HB has also been proven to enhance the physiological activity of cells in previous studies (26). First, the 3HB synthesis has been detected from PHB mobilization based on relevant experiments, and other reports also obtained similar findings in different organisms under various stresses (1). Nevertheless, when we supplied the M1 strain with varying concentrations of 3HB to resist acetic acid stress, the recovery of cell growth was not as robust as observed in the M5 strain. This revealed that the M5 strain’s stress resistance is dictated by the entire PHB mobilization pathway, rather than solely relying on 3HB. In bacterial life processes, PHA synthesis can provide the host with carbon storage, and the PHB granules are also beneficial for cells to resist stress (27). Anyway, PHB degradation is also of great importance, and 3HB is a key intermediate metabolite that can link carbon storage release with cell energy metabolism. Studies have shown that bacteria with an incomplete PHB mobilization system, such as a *phaZ* mutant of *Azospirillum brasilense*, exhibit weaker resistance under various stresses (28). In a word, the resistance of *E. coli* to acetic acid stress by PHB mobilization introduction involves a complex regulatory process, and it will lead to obvious changes in the metabolic mechanism of cells. Thus, the analysis of the global gene transcription level will help clarify the underlying mechanism of the above complex regulatory process.

In this study, the transcriptome sequencing analysis indicated that the introduction of PHB mobilization significantly altered the expression levels of genes involved in cell membrane formation, which plays a critical role in acetic acid stress tolerance. Moreover, RNA-seq analysis of M1 and M5 strains suggested that *Bhsa* of 14 commonly upregulated genes was associated with acetic acid tolerance. BhsA is a protein located on the outer membrane, which plays a critical role in both biofilm formation and stress response (18). Furthermore, previous studies have indicated that overexpression of the *Bhsa* gene can regulate the membrane fluidity of *E. coli* to increase the titer of octanoic acid (29). Notably, the first-time observation revealed that the overexpression of *BhsA* can enhance the resistance of cells to acetic acid. However, this enhancement falls short of the performance achieved by the M5 strain with PHB mobilization. This also indicated that the metabolic regulation of PHB mobilization involves additional function genes, especially those related to cell membranes. Furthermore, the cell membrane is widely recognized as the primary target of damage caused by various environmental stressors, and changes in the composition and function of the cell membrane can affect cell survival during growth under stressful conditions (30). The analysis of the cell membrane revealed that the M5 strain showed improved integrity of the cell membrane under acetic acid pressure, along with a significant reduction in membrane fluidity. Accordingly, the physical properties and physiological functions of lipids and proteins in cell membranes can affect the integrity and fluidity of the membrane, and numerous studies have shown that the functions of cell membranes can be modulated by modifying their molecular composition and structure (31). In this study, we found that PHB mobilization promotes cell resistance to acetic acid by altering the composition of fatty acids. Under acetic acid stress, strain M5 exhibited two changes in its fatty acid composition: a decrease in UFAs and an increase in SFAs and CFAs. According to previous studies, double bonds in UFAs can decrease the order and increase the fluidity of membrane lipids (32). Therefore, a decrease in the proportion of UFAs can reduce membrane fluidity. Besides, previous research has indicated that an increase in CFAs and SFAs can improve the stability of the cell membrane and reduce its fluidity (33). Currently, the manipulation of membrane lipids has proved to be an efficient strategy to enhance membrane function (34). For instance, through the expression of *cis*-*trans* isomerase from *Pseudomonas aeruginosa*, transunsaturated fatty acids were integrated into the membrane of *E. coli*, leading to a reduction in membrane fluidity. This modification led to improvements in acid stress resistance (35). In conclusion, this study indicated that PHB mobilization plays a crucial role in responding to acetic acid stress and preserving cell membrane composition and function.

Interestingly, we further confirmed that the introduction of the PHB mobilization also enhances *E. coli*’s resistance to other acidic stresses (e.g., HCl, lactate, and succinate), indicating that this protective effect is broad spectrum. Previous studies have demonstrated that bacteria inherently harboring PHB mobilization pathway show improved tolerance to a variety of stress conditions (7). In addition, a previous study has demonstrated that the strategy for succinate and PHA co-production is an effective means of generating high-value chemicals (23). In contrast to single-product processes, this integrated production strategy enables more efficient utilization of feedstock and results in fewer accumulated by-products. Hence, employing acetate as the carbon source for microbial fermentation instead of glucose can lead to an additional reduction in co-production costs. However, the toxicity of acetate to cells is responsible for the low utilization efficiency. Thus, PHB mobilization was introduced to improve the cell tolerance to acetate. Then, the genes *phaCABZ* were introduced into strain M6, resulting in strain M8, respectively. Based on the results of fermentation from sodium acetate, strain M8 exhibits better acetate utilization and succinate synthesis ability compared with the recombinant strains without PHB mobilization. This demonstrates that introducing the PHB mobilization pathway confers resistance to acetate stress in the M8 strain, while also significantly enhancing the growth and product synthesis of the cells. Numerous studies have provided evidence that enhancing the robustness of cells can facilitate the production of desired products (36). Notably, the PHB mobilization can generate 3HB, which can still be used for the growth and metabolism of strain M8. Moreover, batch cultivation of the M8 demonstrated that it could simultaneously accumulate extracellular succinate and intracellular PHB. The accumulation of PHB not only increased the yield of succinate but also improved the efficiency of carbon source utilization. Finally, strain M8 can produce 23.93 g/L of succinate by fed-batch fermentation with acetate as the sole carbon source, which was higher than that reported in previous studies (37, 38). Moreover, strain M8 can accumulate 7.21 g/L PHB, and the total yield of the two chemicals can reach 0.69 g/g. These results suggest that this engineered *E. coli* strain has great potential for co-production of succinate and PHB from acetate. In summary, our findings demonstrate that manipulating membrane lipid composition via PHB mobilization introduction could offer a novel approach to enhancing the growth of *E. coli* and the production of high-value chemicals from acetate.

## MATERIALS AND METHODS

### Plasmids, strain construction, and growth conditions

Table 2 presents the strains and plasmids utilized in this study, and standard genetic techniques were used to manipulate all recombinant strains under *E. coli* DH5α background. Plasmids and bacterial genomic DNA were isolated using kits provided by TIANGEN Biotech (Beijing, PR China). Subsequently, the target gene fragments were amplified through polymerase chain reaction. The *phaCABZ* genes and *rpoS* promoter were amplified from the genome of *Cupriavidus necator* and *E. coli*, respectively, to construct recombinant plasmids. The One Step Cloning Kit (Vazyme) was used to insert all fragment products containing homologous sequences into the puc19 plasmid. In addition, mutant strains with gene knockout employed in this study were generated from the *E. coli* DH5α by using the λ red-mediated one-step inactivation method (39). To confirm that the gene of interest had been disrupted, colony PCR and DNA sequencing were carried out on all mutants.

**TABLE 2:** Strains and plasmids used in this study

Strain or plasmid	Relevant characteristics	Reference
Strains		
*E. coli* DH5α	F−, endA1, hsdR17 (rK−, mK+), supE44, thi-l, λ− , recA1, gyrA96	(40)
M1	DH5α derivative, pUC19 plasmids	This study
M2	DH5α derivative, pUC19-*phaA* plasmids	This study
M3	DH5α derivative, pUC19-*phaAB* plasmids	This study
M4	DH5α derivative, pUC19-*phaCAB* plasmids	This study
M5	DH5α derivative, pUC19-*phaCABZ* plasmids	This study
M5-2	DH5α derivative, pUC19- *phaCABZ-2* plasmids	This study
M6	DH5α derivative, Δ*sdhAB*, Δ*iclR*, Δ*maeB*, pUC19 plasmids	This study
M7	M6 derivative, pUC19-*phaCAB* plasmids	This study
M8	M6 derivative, pUC19-*phaCABZ* plasmids	This study
M1-*Bhsa*	DH5α derivative, pUC19-*Bhsa* plasmids	This study
M1-*MtgA*	DH5α derivative, pUC19-*MtgA* plasmids	This study
M1-*ViaA*	DH5α derivative, pUC19-*ViaA* plasmids	This study
M1-*YbjM*	DH5α derivative, pUC19-*YbjM* plasmids	This study
Plasmids		
pTK-Red	*Spc*, *gam-bet-exo* (red recombinase), helper plasmid	(13)
pCP20	*Amp*, *bla*, helper plasmid	(13)
pKD3	*Bla*, FRT-cat-FRT, *rgnB*	(13)
pUC19	pColE1 ori, Amp^R^	(40)
pUC19-*phaA*	pUC19 derivative, *phaA* operon from *C. necator*	This study
pUC19-*phaAB*	pUC19 derivative, *phaAB* operon from *C. necator*	This study
pUC19-*phaCAB*	pUC19 derivative, *phaCAB* operon from *C. necator*	This study
pUC19-*phaCABZ*	pUC19 derivative, *phaCABZ* operon from *C. necator*, *phaZ* under the control of rpoS promoter	This study
pUC19- *phaCABZ-2*	pUC19 derivative, PhaA_C88S_, PhaB_D94A_, PhaC_T323I_, and PhaZ_C183A_ derivatives from *C. necator*, *phaZ* under the control of rpoS promoter	This study
pUC19-*Bhsa*	pUC19 derivative, *Bhsa* from *E. coli*, *Bhsa* under the control of P_J23119_ promoter	This study
pUC19-*MtgA*	pUC19 derivative, *MtgA* from *E. coli*, *MtgA* under the control of P_J23119_ promoter	This study
pUC19-*ViaA*	pUC19 derivative, *ViaA* from *E. coli*, *ViaA* under the control of P_J23119_ promoter	This study
pUC19-*YbjM*	pUC19 derivative, *YbjM* from *E. coli*, *YbjM* under the control of P_J23119_ promoter	This study

The strains used for cloning and inoculation were cultured in LB medium, which consisted of 1% tryptone, 0.5% yeast extract, and 1% NaCl, and were incubated at 37°C for 8–12 hours. The medium was supplemented with antibiotics, including ampicillin (100 mg/L), chloramphenicol (17 mg/L), kanamycin (25 mg/L), and spectinomycin (50 mg/L), as required for the selection of transformed cells. For shake flask fermentation, the mineral salt medium (MSM) was used based on a previous study, and 5 g/L sodium acetate was added (13). A single clone was pre-cultured in 20 mL of LB medium at 37°C with 200 rpm shaking overnight. Then, 5 mL of the overnight culture was used to inoculate 50 mL of MSM for batch fermentation. The flasks were then incubated at 37°C with shaking at 200 rpm, and 50 µg/mL ampicillin was added to the medium to ensure stable expression of transgenes. For fed-batch fermentation in a 5 L fermentor (T&J Bioengineering, Shanghai, China), the culture conditions were the same as in shake flask fermentation. The secondary seed solution was inoculated at 5% into the fermentor, with an initial pH of 7.0. During fermentation, dissolved oxygen was maintained at 30%–50%, pH was controlled at 7.0 with NH_4_OH (25%, vol/vol), and the temperature was set to 37°C. After fermentation, samples are collected for the determination of cell dry weight, carbon source consumption, and analysis of products.

### Spot assays

*E. coli* cells were grown until they reached the logarithmic phase and then diluted to an optical density of 1.0 at 600 nm. Aliquots of 3 µL were taken and subjected to 10-fold serial dilutions. The diluted samples were then spotted onto LB agar plates with or without the specified concentration of acetic acid. The plates were incubated for 2–4 days at 37°C, and the growth of *E. coli* cells was evaluated.

### Growth and survival analysis

To investigate the growth of M5 and M1 strains under the indicated concentration of acetic acid, we diluted logarithmic-phase cells into fresh LB medium with increasing acetic acid concentrations, using an initial OD_600_ of 0.1. To examine the viability of cells, the strains were cultured at various concentrations of acetic acid for 48 hours at 37°C with shaking at 200 rpm. The cells were centrifuged and subsequently washed two times with sterile water, and the OD_600_ values of the final biomass were detected. Then, the data are depicted as a percentage relative to untreated cells of the respective strain, and the half-maximal inhibitory concentration (IC50) was determined by fitting a Hill-type model to the data.

### Transcriptome analysis

Both the M1 and M5 strains were cultured during the logarithmic phase under two different conditions: 0% and 0.06% (vol/vol) acetic acid. Once collected, the strains were frozen at −80°C and sent to the Beijing igeneCode Biotech Co., Ltd for RNA extraction and global gene analysis.

### qRT-PCR analysis

*E. coli* cells were cultured until they reached the logarithmic phase and then harvested by centrifugation at 5,000 × *g* for 2 min at 4°C. Total RNA was extracted using Bacterial RNA Kit (Omega Bio-Tek, Doraville, GA, USA). Subsequently, cDNA was synthesized using the PrimeScript II first-strand cDNA synthesis kit (6210A; TaKaRa Bio). The mRNA level was quantified using SYBR Premix Ex Taq (RR420A; TaKaRa Bio), with 16S rRNA serving as a standard control for normalizing gene expression. Each experiment was repeated three times.

### Membrane fatty acid measurement

The cells of M1 and M5 strains inoculated into fresh LB medium at 0% and 0.06% (vol/vol) acetic acid for 48 hours were used to determine fatty acid composition. Cell samples that had been dried were used for the extraction and methylation of fatty acids, following the previously described procedures (41). The analysis of fatty acids was conducted using a polyethylene glycol capillary column in accordance with previous protocols (42).

### Cell membrane rigidity assay

For membrane rigidity analysis, the *E. coli* cells were treated with 1,6-diphenyl-1,3,5-hexatriene. The spectrofluorometer (Photon Technology International Inc., Edison, NY, USA) was used to determine the steady-state fluorescence anisotropy, and the analysis of the results followed the previously described method (30). Elevated fluorescence anisotropy values generally indicate increased membrane rigidity.

### Detection of 3HB, acetate, succinate, and PHB

For measuring the concentration of acetate, 3HB, and succinate, a high-performance liquid chromatography (HPLC) system (Thermo Fisher Scientific, USA) equipped with a Bio-Rad Aminex HPX-87H ion exclusion particle column (300 × 7.8 mm, Hercules, CA, USA) was used for the analysis. Prior to analysis, the samples were centrifuged at 12,000 rpm for 5 min and filtered through a 0.22 µm aqueous membrane. The HPLC column was operated at a temperature of 65°C using a mobile phase consisting of a 5 mM H_2_SO_4_ solution at a flow rate of 0.6 mL/min. To quantify the PHB concentration, 50 mg of freeze-dried cells was added to an esterification flask containing 2 mL of chloroform, 850 µL of methanol, and 150 µL of concentrated sulfuric acid, and the mixture was heated at 100°C for 1 hour. After cooling, 1 mL of distilled water was added, and the chloroform phase was separated from the lower layer for analysis. The sample was analyzed using a gas chromatograph (Shimadzu GC-2010) with a 30 m × 0.32 mm Rtx-5 column. Nitrogen gas was used as the carrier at a flow rate of 3.0 mL/min, and the inlet temperature was set to 210°C. The column oven temperature was initially held at 80°C for 5 min, then ramped to 220°C at a rate of 20°C/min, and finally maintained at 220°C for 5 min. A flame ionization detector at 275°C was used for peak detection. The PHB concentration was determined by quantifying the monomer methyl-3-hydroxybutyrate, with methyl-3-hydroxybutyrate (Sigma-Aldrich, Shanghai, China) used as a standard to calculate the PHB concentration (g/L), following a previously described method (43).

### Fourier transform infrared spectroscopy analysis

Fourier transform infrared spectroscopy has been extensively utilized to analyze the alterations in the structure of bacterial cell membranes under various stress conditions. The samples were analyzed using FTIR in accordance with a previously described method, and the FTIR spectrum of each pellet was recorded at 400–4,000 cm^−1^ (21).

### Assessment of cell membrane integrity

The assessment of cell membrane damage was conducted using the fluorescent dye propidium iodide according to a previous study. The cells of M1 and M5 strains in the logarithmic growth phase were inoculated into fresh LB medium at 0% and 0.06% (vol/vol) acetic acid for 12 hours. Samples were centrifuged, washed twice with PBS, and diluted to an OD_600_ of 0.5. To monitor the integrity of the cell membrane, 500 µL diluted samples were incubated with 3 µL of propidium iodide (Sigma, Shanghai City, China) at a concentration of 1 mg/mL. The mixtures were rapidly homogenized and incubated in the dark at room temperature for 5 min and placed on a microscope slide covered with a coverslip. Then, the samples were subjected to confocal fluorescence microscopy (Olympus FV1000) analysis. In addition, the mixtures were also used for the evaluation of PI uptake. The fluorescence was measured with a fluorimeter (Model LS-5B, PerkinElmer, MA, USA) at an excitation wavelength of 495 nm and an emission wavelength of 615 nm, and the data were processed based on the previous report (44).

## Data Availability

The RNA-seq raw reads were deposited in the NCBI under the Sequence Read Archive (SRA) accession number PRJNA1206825. Additional data supporting the plots and findings of this study are available from the corresponding author upon reasonable request.

## SUPPLEMENTAL MATERIAL

Supplemental figures aem.02454-24-s0001.docx Figures S1 to S3.

ASM does not own the copyrights to Supplemental Material that may be linked to, or accessed through, an article. The authors have granted ASM a non-exclusive, world-wide license to publish the Supplemental Material files. Please contact the corresponding author directly for reuse.
