# Supplementary material for: Improvement of acetate tolerance of Escherichia coli by introducing the PHB mobilization pathway
Source: Appl Environ Microbiol. 2025 Apr 4;91(5):e02454-24. doi: 10.1128/aem.02454-24 (PMC12093944; doi:10.1128/aem.02454-24)
Supplement: Supplemental figures — Figures S1 to S3. [file aem.02454-24-s0001.docx]

**Supplementary Material**

**Improvement of acetate tolerance of *Escherichia coli* by introducing the PHB mobilization pathway**

Dong Meng^a^, Shuai Wang^a^, Ke Zhao ^b^, Yan Luo^a^, Xu Li^a^, Ying Wang^a^*

^a^ Key Laboratory of Medical Molecule Science and Pharmaceutical Engineering, Ministry of Industry and Information Technology, Institute of Biochemical Engineering, Department of Chemical Engineering, School of Chemistry and Chemical Engineering, Beijing Institute of Technology, Beijing, 100081, China

^b^ MOE Key Laboratory of Cluster Science, Beijing Key Laboratory of Photoelectronic/Electrophotonic Conversion Materials, School of Chemistry and Chemical Engineering, Beijing Institute of Technology, Beijing, 100081, China

* Corresponding author: Ying Wang

E-mail address: wy2015@bit.edu.cn


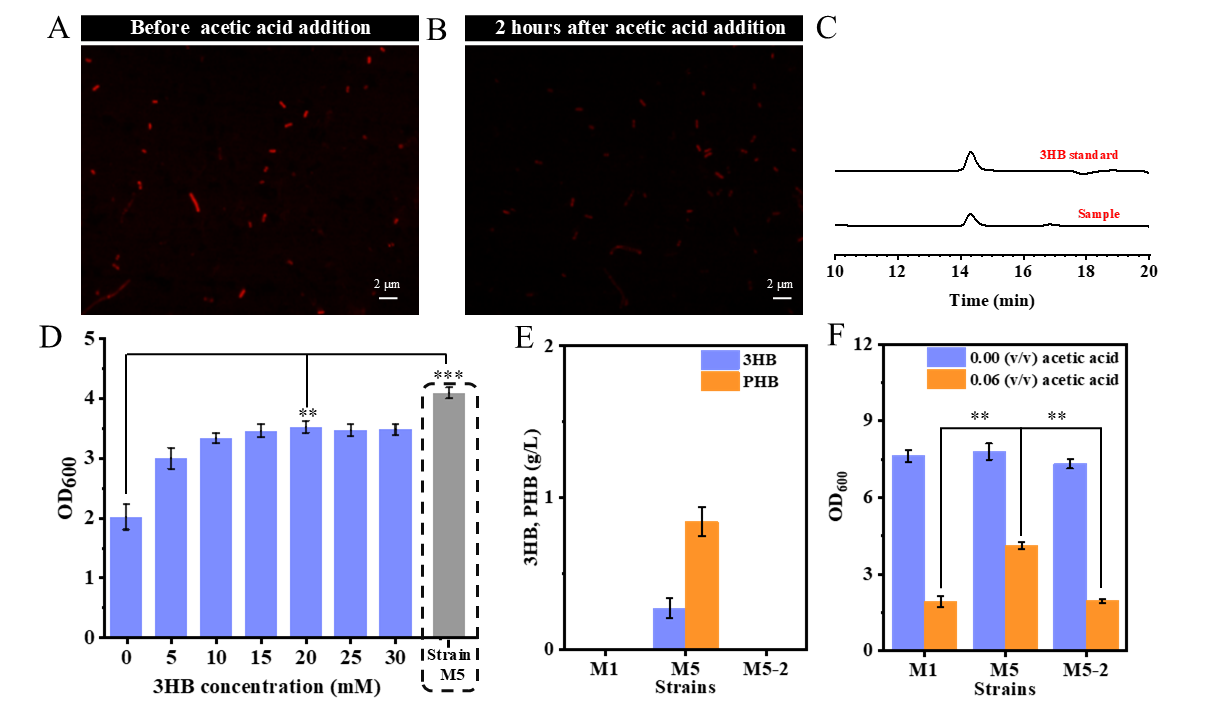


**FIG. S1.** PHB mobilization contributes to acetic acid resistance. (A and B) Fluorescent microscope images of M5 strain before and after acetic acid addition. (C) HPLC analysis of 3HB. (D) the final OD_600_ of M1 fed with various concentrations of 3HB. (E) The 3HB and PHB concentration of strains M1, M5, and M5-2 at 0.06% (v/v) acetic acid (F) The final OD_600_ of strains M1, M5, and M5-2 at 0% and 0.06% (v/v) acetic acid. *P ≤ 0.05, **P ≤ 0.01 ***P ≤0.001


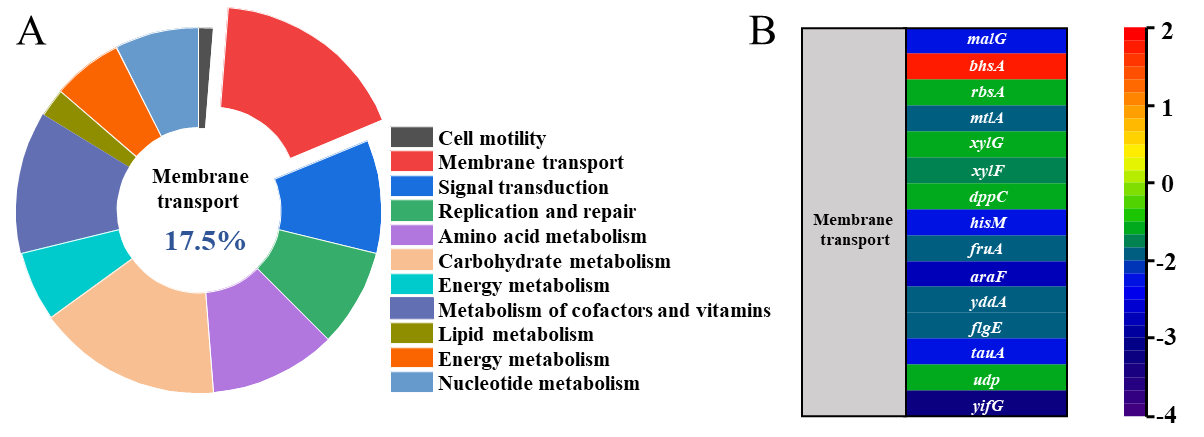


**FIG. S2.** Transcriptome analysis of the strains M1 and the M5 at 0.06% (v/v) acetic acid. (A) Comparison of differentially expressed genes between the M1 and the M5 strains as annotated using the KEGG database. (B) Heat map of the [membrane transport](javascript:;) module at 0.06% (v/v) acetic acid following comparison between the strains M1 and the M5.


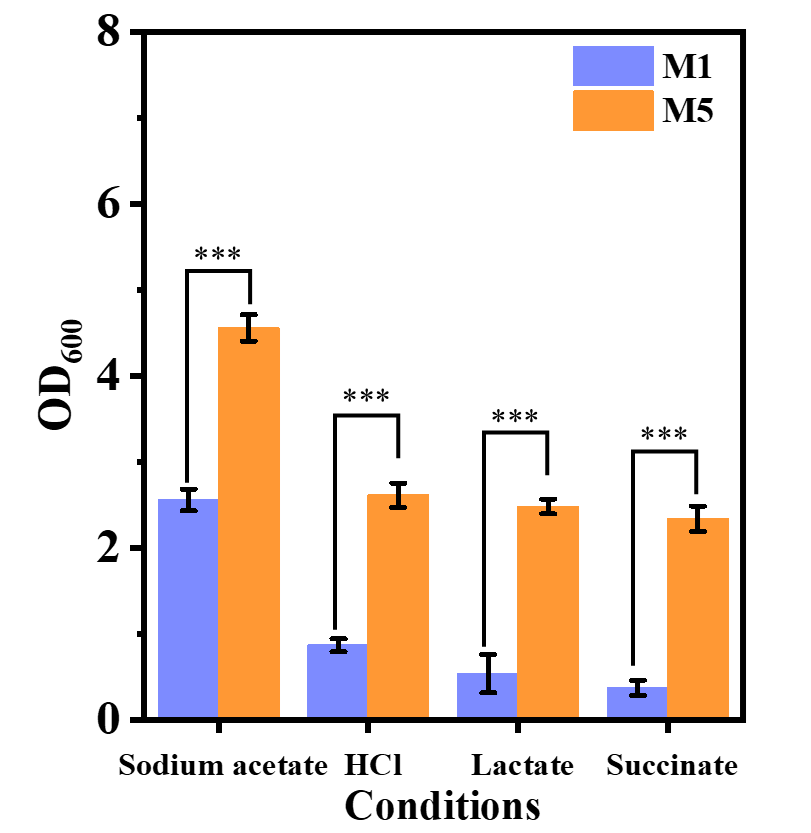


**FIG. S3.** The final OD_600_ of the strains M1 and M5 after 48 hours under various stress conditions. Sodium acetate: 5 g/L; HCl: 0.01% (v/v); Lactate: 5 g/L; Succinate: 30 g/L. *P ≤ 0.05, **P ≤ 0.01 ***P ≤0.001
